# Analysis of intracranial lesions in patients with HIV-associated cryptococcal meningitis

**DOI:** 10.3389/fcimb.2025.1446470

**Published:** 2025-02-03

**Authors:** Wei Song, Li Liu, Tangkai Qi, Zhenyan Wang, Yang Tang, Jianjun Sun, Shuibao Xu, Junyang Yang, Jiangrong Wang, Jun Chen, Renfang Zhang, Yinzhong Shen

**Affiliations:** Department of Infection and Immunity, Shanghai Public Health Clinical Center, Fudan University, Shanghai, China

**Keywords:** cryptococcal meningitis, HIV, intracranial lesions, cerebrospinal fluid, cytokines

## Abstract

**Background:**

Intracranial imaging abnormalities are commonly observed in patients suffering from HIV-associated cryptococcal meningitis, both before and during the treatment period. This study aims to analyze the prevalence, origins, radiological characteristics, treatments, and prognosis of intracranial lesions in patients with HIV-associated cryptococcal meningitis, thereby providing references for future clinical decision-making.

**Methods:**

The clinical data of patients diagnosed with HIV-associated cryptococcal meningitis and admitted to the Shanghai Public Health Clinical Centre between 2013 and 2019 were collected. Logistic regression analysis was subsequently conducted to identify potential risk factors associated with the development of intracranial lesions in this patient group.

**Results:**

Of 211 patients analyzed, 64.5% (136/211) had intracranial lesions during treatment and follow-up. Initial cranial imaging showed 60% had lesions pre-treatment. Throughout treatment, 32.7% (52/159) developed new or worsened lesions. Mortality rates at 2 weeks, 8 weeks, and 2 years for those with detected lesions were 3%, 7.6%, and 13.2%, respectively. Lesions were primarily caused by *Cryptococcus* (70.5%) and *Mycobacterium* (24.3%). Lacunar infarcts, especially in the basal ganglia, were the most common type. Patients aged 50 years or older, and those presenting with altered mental status upon admission, were found to be more likely to have intracranial lesions at baseline, with adjusted odds ratios of 5.364 (95% CI: 1.468-19.591, P=0.011) and 7.970 (95% CI: 2.241-28.337, P=0.001), respectively. Patients with lesion progression showed higher levels of IFN-γ, IL-4, IL-5, IL-6, IL-1Ra, IL-1β, GM-CSF, Eotaxin, and Basic FGF in cerebrospinal fluid after four weeks of treatment.

**Conclusion:**

Intracranial lesions in HIV-associated cryptococcal meningitis patients are mostly due to Cryptococcus and Mycobacterium infections. They often appear as lacunar infarcts, predominantly in the basal ganglia, and can worsen with treatment initiation, possibly due to higher baseline cytokine levels in cerebrospinal fluid.

## Introduction

Cryptococcal meningitis, an infection of the central nervous system caused by the fungus *Cryptococcus*, stands as one of the prevalent opportunistic infections affecting individuals living with human immunodeficiency virus (HIV) (Rajasingham et al., 2022). Currently, it remains a leading cause of HIV-related mortality, with reported fatality rates ranging from 18.9% to 47% within 8 to 10 weeks after the onset of illness (Song et al., 2020; Day et al., 2013; Beardsley et al., 2016; Molloy et al., 2018). Intracranial imaging abnormalities are notably common in HIV-positive individuals diagnosed with cryptococcal meningitis, both at the outset of therapy and throughout its course. Previous summaries have indicated that a concerning 67.6% of these patients display such abnormalities upon initial diagnosis and admission for treatment, while 29.4% undergo the emergence or exacerbation of intracranial lesions during the therapeutic process (Song et al., 2020).

In patients diagnosed with cryptococcal meningitis, intracranial lesions are primarily attributed to the cryptococcal infection itself. Intracranial abnormalities observed in the context of HIV-associated cryptococcal meningitis may manifest as various presentations, including intracranial masses, dilated perivascular spaces, cortical and lacunar infarcts, pseudocysts, hydrocephalus, as well as encephalitis and meningitis (Charlier et al., 2008; Katchanov et al., 2015; Offiah and Naseer, 2016; Loyse et al., 2015). Studies have indicated that intracranial masses constitute 4.6% to 21% of observed lesions (Chen et al., 2000; Kure et al., 1991; Charlier et al., 2008; Choe et al., 2010), while lacunar infarcts are reported to occur in up to 20% of cases, with cortical infarcts being similarly frequent. These ischemic changes typically affect the small penetrating branches of the cerebral arteries (Katchanov et al., 2015; Loyse et al., 2015).

Moreover, in the setting of HIV-induced immunodeficiency, intracranial lesions in patients with HIV-associated cryptococcal meningitis may also stem from other opportunistic infections and tumors. Prior research highlights toxoplasmosis as the primary pathological cause of such lesions in HIV-positive individuals, followed by primary central nervous system lymphoma. Additional etiologies include *Mycobacterium tuberculosis*, syphilis, cytomegalovirus, JC virus infections, and histoplasmosis (Smego et al., 2006; d'Arminio Monforte et al., 2004). Therefore, accurate differentiation and identification of the underlying nature of intracranial lesions in HIV-infected individuals with cryptococcal meningitis are crucial in clinical practice.

Previous studies have suggested that up to 30% of patients with HIV-associated cryptococcal meningitis may develop IRIS within weeks or months following the initiation of antiretroviral therapy (ART), characterized by a worsening of clinical symptoms (Lortholary et al., 2005; Müller et al., 2010). This syndrome is hypothesized to result from an abnormal inflammatory reaction to residual cryptococcal antigens during the recovery of the host’s immune function (French, 2009). Central nervous system space-occupying lesions, consistent with the diagnostic criteria for HIV-related cryptococcal meningitis IRIS (Haddow et al., 2010), typify this clinical syndrome. Following the exclusion of alternative etiologies, the emergence of new or progressive intracranial lesions concurrent with clinical deterioration, as observed in our previous research during the post-ART treatment phase, is attributed to IRIS (Song et al., 2020).

The treatment of HIV-associated cryptococcal meningitis presents numerous challenges, and the presence of intracranial lesions further complicates diagnosis and management. Hence, it is vital to identify the characteristics and etiology of intracranial lesions in these patients to improve clinical outcomes and prognosis. To this end, we analyzed intracranial lesions in patients with HIV-associated cryptococcal meningitis, summarizing the prevalence, causes, radiological features, subsequent treatments, and prognostic factors, with the aim of providing references for future clinical decision-making.

## Methods

### Patients and samples

This study investigated all instances of HIV-associated cryptococcal meningitis cases admitted to the Shanghai Public Health Clinical Center (SPHCC) from January 2013 to December 2019. It included patients who were undergoing their initial diagnosis and treatment for cryptococcal meningitis, whether they were newly diagnosed at our institution or transferred from another medical facility for ongoing care. Patient outcomes at the two-year mark were verified through telephone follow-up.

All enrolled patients underwent a blood test to confirm HIV positivity via Western blot, and they exhibited cerebrospinal fluid (CSF) positivity for C. neoformans, confirmed by either a positive India ink staining result or fungal culture.

### Etiological diagnosis of intracranial lesions

Intracranial lesions are identified as abnormalities through cranial magnetic resonance imaging (MRI) or computed tomography (CT) scans. Diagnoses of conditions such as lacunar infarctions are formally established by radiologists in official imaging reports.

This study conducted a retrospective collection of clinical data throughout the patients’ treatment and follow-up processes, combining it with final prognoses to classify the etiology of intracranial lesions. For instance, some patients showed positive acid-fast staining, or Mycobacterium culture or PCR positivity in their CSF, and were thus classified as having direct pathogenic evidence from the central nervous system (CNS). Meanwhile, other patients did not have identifiable pathogens in their CSF, with diagnoses relying on other indirect evidence or solely on clinical judgment by physicians. The etiology of intracranial lesions were classified into the following categories:

1. Attributable to Infection with *Mycobacterium* (treated with anti-mycobacterial therapy): a. Supported by direct evidence from the CNS; b. In the absence of direct CNS evidence, but evidence of pathogenesis found in specimens from other sites; c. Absent pathogenic evidence, the diagnosis of Mycobacterial infection was made based on pulmonary imaging findings, which was further determined to be the etiology of the intracranial lesion; d. Diagnosed predominantly through clinical observations and characteristics of the CSF.
2. Related to *Cryptococcus*: e. Due to cryptococcal immune reconstitution syndrome, management included the additional administration of steroids and thalidomide, in conjunction with antifungal treatment. f. Cryptococcal infection-related, treatment was confined to the use of antifungal medications. g. Cryptococcal infection -related, but complicated by extracerebral Mycobacterium infection.
3. Other Causes

It is significant to note that in cases of cryptococcal meningitis, it is not always possible to fully exclude the impact or consequences of central cryptococcal infections in intracranial lesions attributed to non-cryptococcal causes.

### Treatment scheme

All patients received treatment through an intravenous infusion of the conventional amphotericin B (at a dosage of 0.5 mg/kg/day), which was the only amphotericin B formulation accessible at our center. This treatment was combined with an oral induction course of flucytosine (5-FC; 100 mg/kg/day) for a minimum duration of four weeks. Following the induction phase, an oral consolidation regimen of Fluconazole at 400 mg daily was administered until the CSF India ink staining tests returned negative results. Thereafter, a maintenance regimen of Fluconazole at 200 mg daily was continued until the patient’s CD4 count surpassed 100 cells/mm³. The duration of the antifungal therapy for all patients exceeded one year due to the unavailability of alternative amphotericin B formulations at SPHCC; consequently, no substitute treatments could be offered.

For patients who had commenced ART prior to being diagnosed with cryptococcal meningitis, the therapy was ongoing. In contrast, ART initiation for ART-naive patients was postponed to four weeks following the initiation of antifungal treatment.

Upon admission, all patients were subject to a lumbar puncture, executed either before or after the administration of mannitol, rendering initial intracranial pressure measurements incomparable. The frequency of mannitol administration was tailored based on clinical needs, ranging from three to six times a day. During the hospital stay, lumbar punctures were performed approximately on a weekly basis, contingent upon clinical indicators. Following discharge, patients were scheduled for lumbar punctures every three months as a part of their follow-up protocol. Most patients underwent baseline cranial imaging, primarily MRI or CT scans, with subsequent scans being conducted as clinically warranted.

In cases where intracranial lesions were diagnosed as stemming from *Mycobacterium tuberculosis*, the medication regimen included rifampin (or Rifabutin), isoniazid, ethambutol, and pyrazinamide. For lesions associated with non-tuberculous Mycobacterium infections, the treatment regimen comprised azithromycin (or clarithromycin), rifabutin (or rifampin), ethambutol, and either levofloxacin or moxifloxacin. Alternatively, a combined treatment approach was implemented, integrating rifampin (or rifabutin), isoniazid, ethambutol, pyrazinamide, azithromycin (or clarithromycin), along with levofloxacin or moxifloxacin.

The application of corticosteroids and/or thalidomide was based on the specific disease state to alleviate intracranial inflammation. Patients suspected to be experiencing cryptococcal IRIS were administered corticosteroids and/or thalidomide in conjunction with the ongoing antifungal therapy, as deemed appropriate by clinical judgment.

### CSF cytokine assay

We gathered CSF residue samples from a subset of patients before the start of treatment and at 1, 2, 3, and 4 weeks after initiating the treatment. These samples were then preserved at -80 degrees Celsius in a freezer. Following this preservation, we performed an analysis of 27 cytokines using the Luminex multiplexing technology (FLEXMAP 3D system) in combination with the Bio-Plex Pro Human Cytokine 27-Plex Assay Kit (Catalog Number M500KCAF0Y).

### Outcome assessment

In this study, we meticulously detail the occurrence rates of intracranial lesions in these patients, highlighting their radiological characteristics. Moreover, we have undertaken a systematic analysis and cataloging of the etiologies responsible for these lesions. This is coupled with a concise summary of the treatment methodologies employed and their subsequent prognoses. Our investigation primarily focuses on identifying factors associated with the development of intracranial lesions, taking the patients’ initial clinical features upon admission as the foundational baseline. Furthermore, we examine whether there are significant differences in the levels of CSF cytokines among patients who experience a deterioration of intracranial lesions during their treatment course.

### Statistical analysis

We executed statistical evaluations utilizing SPSS version 25.0. Quantitative datasets were delineated as medians accompanied by interquartile ranges, while categorical datasets were encapsulated as percentages. For the purpose of discerning disparities amongst groups, chi-square tests or Fisher’s exact test were applied as deemed suitable, in conjunction with logistic univariate regression analysis. Variables that were identified as statistically significant (P < 0.05) during the univariate analysis were then incorporated into a logistic multivariate regression model to further ascertain their impacts. To evaluate variances in CSF cytokine concentrations across patient subgroups, categorized by unique clinical attributes, the Mann–Whitney U test was deployed.

## Results

### Prevalence and prognosis of intracranial lesions

A total of 229 cases of HIV-associated cryptococcal meningitis were recorded at SPHCC from the years 2013 to 2019. After excluding 18 patients without cranial imaging data, 211 cases were included in the study.

During the entire treatment and follow-up period, it was observed that 64.5% (136 out of 211) of patients manifested concurrent intracranial lesions as evidenced by abnormalities in cranial imaging. Specifically, at baseline assessment, 60% (108 out of 180) were found to have intracranial lesions. Moreover, 32.7% (52 out of 159) experienced either the emergence of new lesions or a worsening of pre-existing lesions over the course of treatment. Significantly, the progression of intracranial lesions was noted only after the negativization of CSF Crytococcus cultures and the initiation of ART. Furthermore, once the initial CSF *Cryptococcus* culture became negative, no subsequent positive cultures were detected in the follow-up evaluations.

Over the course of treatment and follow-up, the all-cause mortality rates among patients with intracranial lesions were observed to be 3% (4 out of 135) at 2 weeks, 7.6% (10 out of 131) at 8 weeks, and 13.2% (16 out of 121) at the 2-year mark. Notably, for patients who either developed new lesions or experienced deterioration of existing lesions during treatment, the 2-year all-cause mortality rate was 9.1% (4 out of 44) (**Table 1**).

**Table 1:** Outcomes of patients.

	Outcomes of 2 weeks n (%)	Outcomes of 8 weeks n (%)	Outcomes of 2 years n (%)
All patients (n=229)			
Survivors Deaths Patients lost to follow-up	205 (91.1) 20 (8.9) 4	187 (86.2) 30 (13.8) 12	170 (82.1) 37 (17.9) 22
Patients with cranial imaging data (n=211)			
Survivors Deaths Patients lost to follow-up	200 (95.7) 9 (4.3) 2	183 (90.6) 19 (9.4) 9	167 (86.5) 26 (13.5) 18
Patients with intracranial lesions throughout the entire treatment and follow-up (n=136)			
Survivors	131 (97)	121 (92.4)	105 (86.8)
Deaths	4 (3)	10 (7.6)	16 (13.2)
Patients lost to follow-up	1	5	15
Patients with new intracranial lesions or worsening of original lesions during treatment (n=52)			
Survivors	52	52	40 (90.9)
Deaths	0	0	4 (9.1)
Patients lost to follow-up	0	0	8

### Disease spectrum analysis of intracranial lesions

Throughout the treatment and follow-up of 136 patients with intracranial lesions, 33 (24.3%) were due to Mycobacterial infections, and 96 (70.5%) were connected to cryptococcal infections. Additionally, 7 cases (5.1%) were caused by various other factors: 2 instances of Toxoplasma infection, 1 unspecified malignant tumor, 1 herpes virus infection, 1 brain abscess leading to the patient’s demise before a specific cause could be identified, 1 lymphoma, and 1 case of progressive multifocal leukoencephalopathy (**Table 2**).

**Table 2:** Disease spectrum analysis of intracranial lesions.

Possible causes of intracranial lesions	n (%)
**The intracranial lesions that have appeared throughout the entire course of treatment and follow-up**	**136 (100)**
** Mycobacterium infection-related**	33 (24.26)
Pathogenological evidence found in CSF	5 (3.68)
Evidence of pathogenesis found in specimens from other sites	7(5.15)
Considering possibility of mycobacterial infection based on pulmonary imaging findings	10 (7.35)
Diagnosis made based solely on the patient’s clinical presentation and CSF characteristics	11 (8.09)
**Cryptococcal infection-related**	96 (70.59)
Cryptococcal meningitis IRIS related, with steroid therapy being used	7 (5.15)
Cryptococcal infection-related, only with antifungal therapy being used	75 (55.15)
Cryptococcal-related, but complicated by extracerebral Mycobacterium infection	14 (10.29)
** Other causes**	7 (5.15)
**New lesions or existing lesions worsened during treatment**	**52 (100)**
** Mycobacterium infection-related**	28 (53.85)
Pathogenological evidence found in CSF	3 (5.77)
Evidence of pathogenesis found in specimens from other sites	6 (11.54)
Considering possibility of mycobacterial infection based on pulmonary imaging findings	9 (17.31)
Diagnosis made based solely on the patient’s clinical presentation and CSF characteristics	10 (19.23)
** Cryptococcal infection-related**	21 (40.38)
Cryptococcal meningitis IRIS related, with steroid therapy being used	7 (13.46)
Cryptococcal infection-related, only with antifungal therapy being used	9 (17.31)
Cryptococcal-related, but complicated by extracerebral Mycobacterium infection	5 (9.62)
** Other causes**	3 (5.77)

The bolded sections serve as the higher-level categories for prominent display.

In analyzing the medical records of 52 patients who developed new lesions or witnessed an exacerbation of pre-existing ones during their treatment course, it was noted that 28 cases (53.8%) were clinically inferred to be due to Mycobacterial infections, with each patient undergoing appropriate treatment for Mycobacteria. Etiological proof of Mycobacterial infection was established in 9 cases (17.3%), with the presence of Mycobacteria confirmed in central or extracentral sites. Conversely, cryptococcal infections were implicated in 21 cases (40.4%), including 7 cases (13.5%) recognized as C-IRIS. This diagnosis was made when imaging displayed mass lesions, which were subsequently verified by surgical biopsy pathology. Post-treatment observations showed an improvement in symptoms and a reduction in lesion size following the administration of steroids in conjunction with ongoing antifungal therapy (**Table 2**).

### Imaging characteristics of intracranial lesions

After the exclusion of cases with incomplete imaging data, an analysis was conducted on the lesion locations for 94 patients who exhibited cranial imaging abnormalities initially and for 49 patients who either manifested new lesions or experienced worsening of pre-existing ones during their treatment course (**Table 3**). Radiological evaluations revealed that 42 (44.7%) of the initial patient group had suffered lacunar infarcts. Within this group presenting initial cranial imaging deviations, the most commonly affected areas were the basal ganglia (40.4%), the frontal lobe (37.2%), and the ventricles along with the periventricular zones (27.7%), with the parietal lobe following closely (26.6%). For the patients experiencing progression of intracranial lesions during treatment, the primary sites susceptible to lesion development, in order, were the parietal lobe (57.1%), frontal lobe (51%), basal ganglia (32.7%), and the occipital lobe (32.7%).

**Table 3:** The location of intracranial lesions.

Location	n (%) Intracranial lesions at baseline n=94 94 (100)	n (%) New lesions or existing lesions worsened during treatment 49 (100)
Around the ventricles and lateral ventricles Basal ganglia Frontal lobe Parietal lobe Occipital lobe Cerebellum Temporal lobe Corona radiata Sub-cortex Midbrain Centrum semiovale	26 (27.7) 38 (40.4) 35 (37.2) 25 (26.6) 11 (11.7) 11 (11.7) 12 (12.8) 10 (10.6) 10 (10.6) 4 (4.3) 5 (5.3)	14 (28.6) 16 (32.7) 25 (51.0) 28 (57.1) 16 (32.7) 14 (28.6) 12 (24.5) 6 (12.2) 3 (6.1) 1 (2.0) 5 (10.2)

### Treatment of intracranial lesions

For patients who develop new intracranial lesions or experience exacerbation of existing ones during therapy, we have compiled their therapeutic strategies. While continuing to maintain antifungal therapy for *Cryptococcus*, the management of these intracranial lesions primarily involves anti-mycobacterial therapy along with steroid-based anti-inflammatory treatment, occasionally supplemented with thalidomide. Noteworthy is the fact that, among seven individuals exhibiting intracranial lesions attributable to cryptococcal immune reconstitution syndrome, remission was achieved solely through the administration of corticosteroids (with two cases also involving thalidomide) (**Table 4**).

**Table 4:** Treatment of Intracranial Lesions.

Possible causes of intracranial lesions	Medication regimen	n	Duration of Mycobacterium treatment (months) Median (interquartile range)	Cases using glucocorticoids n (%)	Cases using thalidomide n (%)
Mycobacterium infection-related	Regimens for Anti-Mycobacterium (Total)	29	18 (17.50-20.00)	25 (86.2)	6 (20.7)
	rifampin (or rifabutin), isoniazid, ethambutol, and pyrazinamide	6	18 (11.25-19.25)	6 (100)	1 (16.7)
	azithromycin (or clarithromycin), rifabutin (or rifampin), ethambutol, and either levofloxacin or moxifloxacin	9	18 (18.00-22.00)	5 (55.6)	2 (22.2)
	rifampin (or rifabutin), isoniazid, ethambutol, pyrazinamide, azithromycin (or clarithromycin), along with levofloxacin or moxifloxacin.	14	18 (16.25-20.50)	14 (100)	3 (21.4)
Cryptococcal meningitis IRIS related	corticosteroids and/or thalidomide in conjunction with the ongoing antifungal therapy	7		7 (100)	2 (28.6)

Of the 29 patients suspected of having intracranial mycobacterial infections, six were diagnosed with tuberculosis and showed a favorable response to the treatment; nine were believed to have non-tuberculous mycobacterial infections. Among these, one patient regrettably passed away five months into treatment, whereas the remaining patients generally had a positive outcome. For 14 patients, clinical judgment necessitated a dual therapeutic approach due to diagnostic ambiguity, aiming to target both tuberculous and non-tuberculous mycobacteria. Within this cohort, one patient’s condition worsened, leading to their demise two years into treatment, while the others demonstrated notable improvements.

### Analysis of factors related to intracranial lesions

After performing a univariate logistic regression analysis on the initial clinical characteristics of the patients, we discovered that individuals aged 50 years and above, those of the female gender, and those exhibiting altered consciousness upon admission were linked with the presence of concurrent intracranial lesions at baseline. Advancing to a multivariate logistic regression analysis with these three variables revealed that being aged 50 years and above (Adjusted Odds Ratio [AOR] 5.364, 95% Confidence Interval [CI] 1.468-19.591, *P*=0.011) and having altered consciousness at the time of admission (AOR 7.970, 95% CI 2.241-28.337, *P*=0.001) remained significantly as independent predictors for the existence of baseline intracranial lesions (**Table 5**).

**Table 5:** Associations between clinical characteristics and intracranial lesions.

Variables	n	Patients with intracranial lesions (%)	OR (95% CI), Univariable	*P*	AOR (95%CI), Multivariable	*P*
Age						
< 50 years ≥50 years	153 27	85 (55.6) 23 (85.2)	1 4.600 (1.518–13.939)	0.007	1 5.364 (1.468–19.591)	0.011
Sex						
Female Male	15 165	13 (86.7) 95 (57.6)	1 0.209 (0.046–0.955)	0.043	1 0.271 (0.054–1.367)	0.114
ART status						
ART-naïve ART-treated	153 27	95 (62.1) 13 (48.1)	1 0.567 (0.249–1.290)	0.176		
ART status						
< 50 cells/μl ≥50 cells/μl	148 23	86(58.1) 15(65.2)	1 1.352 (0.540–3.385)	0.520		
Hemoglobin						
≥90 g/L < 90 g/L	138 10	81 (58.7) 8 (80)	1 2.815 (0.576–13.749)	0.201		
CSF white cell count						
< 20 cells/mL ≥20 cells/mL	105 43	61 (58.1) 28 (65.1)	1 1.346 (0.644–2.814)	0.429		
CSF protein						
< 500 mg/L ≥500 mg/L	94 53	54 (57.4) 34 (64.2)	1 1.326 (0.662–2.655)	0.426		
CSF glucose						
≥2.0 mmol/L < 2.0 mmol/L	95 51	53 (55.8) 36 (70.6)	1 1.902 (0.920–3.930)	0.083		
Time interval between onset and diagnosis						
< 14 days ≥14 days	67 81	37 (55.2) 52 (64.2)	1 1.454 (0.750–2.818)	0.268		
Altered mental status on admission						
No Yes	119 29	63 (52.9) 26 (89.7)	1 7.704 (2.211–26.838)	0.001	1 7.970 (2.241–28.337)	0.001

OR, Odds ratio; AOR, Adjusted odds ratio; CI, Confidence interval; ART, Antiretroviral therapy; CSF, Cerebrospinal fluid.

Characteristics of CSF Cytokines in Patients with Progressive Intracranial Lesions

A total of 48 CSF samples were collected at various time points from patients, which included 38 samples before treatment initiation, 38 samples one week into the treatment, 28 samples at the end of the second week, 18 samples three weeks into treatment, and 21 samples after four weeks of treatment. Only patients with complete imaging records were selected for this study and were further categorized based on the progression status of their intracranial lesions following the treatment. A comparative analysis was conducted on the CSF cytokine levels at each time point between the two groups. The analysis revealed that, at the four-week mark of treatment, patients experiencing progression in their intracranial lesions had significantly higher levels of IFN-γ, IL-4, IL-5, IL-6, IL-1Ra, IL-1β, GM-CSF, Eotaxin, and Basic FGF in their CSF compared to patients without lesion progression (**Table 6**). It is important to note, however, that no significant differences in cytokine levels were observed between the two groups at the earlier time points under investigation.

**Table 6:** Comparison of CSF cytokines between two groups of patients after four weeks of treatment.

CSF cytokines at 4-week (pg/ml)	No progression of intracranial lesions after treatment (*n=9*)	New lesions or existing lesions worsened (*n=8*)	*P*
IFN-γ	0.84 (0.58-1.45)	2.30 (1.51-4.10)	0.006
IL-4	0.13 (0.11-0.16)	0.19 (0.16-0.26)	0.021
IL-5	7.00 (4.49-13.79)	17.01 (11.63-15.65)	0.015
IL-6	2.45 (1.97-4.98)	5.12 (3.46-73.86)	0.036
IL-1Ra	300.9 (52.6-840.9)	756.9 (598.1-1443.5)	0.046
IL-1β	0.11 (0.10-0.14)	0.18 (0.14-0.20)	0.008
GM-CSF	0.14 (0.11-0.16)	0.21 (0.15-0.25)	0.036
Eotaxin	0.69 (0.60-0.88)	0.95 (0.87-1.11)	0.021
Basic FGF	2.91 (2.80-3.66)	4.63 (3.50-5.97)	0.006

Continuous variables are expressed as the median (interquartile range, IQR). IFN-γ, interferon gamma; IL-4, interleukin-4; IL-5, interleukin-5; IL-6, interleukin-6; IL-1Ra, interleukin-1 receptor antagonist; IL-1β, Interleukin-1 beta; GM-CSF, granulocyte-macrophage colony-stimulating factor; Eotaxin, eosinophil chemotactic protein; Basic FGF, basic fibroblast growth factor.

## Discussion

This investigation disclosed that intracranial lesions are common, with a prevalence of 64.5%, in patients suffering from HIV-associated cryptococcal meningitis. This condition complicates the clinical management and challenges the development of optimal therapeutic strategies. The cohort under study identified the cryptococcal infection as the primary cause of such lesions. It is crucial to recognize that various opportunistic infections due to AIDS-induced immunodeficiency may similarly impact the CNS. Against this backdrop, the research embarked on a comprehensive review of the causes behind intracranial lesions in patients positive for HIV with cryptococcal meningitis. Our retrospective study did not solely depend on in-depth etiological test outcomes for diagnosis clarification but also used the therapeutic response to medications as an inference tool in cases with undetermined etiology. Hence, the range of intracranial lesions documented in this study was reliable and provides valuable reference data. Our analysis indicates that 70.5% of the lesions observed during the treatment of patients were linked to cryptococcal infection. Given the involvement of *Cryptococcus* in the CNS in all studied subjects, lesions that appeared unrelated may also have been influenced by the cryptococcal activity, suggesting the actual proportion related to cryptococcal infection could be higher. This insight emphasizes the importance of considering cryptococcal infection as the primary diagnosis in similar clinical scenarios. Nevertheless, we also found mycobacterial infections in 24.3% of patients, underscoring its significance as another prevalent condition in AIDS patients warranting early consideration in the diagnostic process.

Cryptococcal infection has been implicated as a causal factor in ischemic strokes (Fugate et al., 2014). The occurrence of ischemic stroke among individuals with cryptococcal meningitis varies widely, with prevalences reported from 13% to 54% across numerous studies (Mishra et al., 2018; Chen et al., 2011b; Lan et al., 2001; Qu et al., 2017). It is not uncommon for patients to first present with symptoms of a stroke before being diagnosed with cryptococcal meningitis (Shimoda et al., 2017; Aharon-Peretz et al., 2004; Saul et al., 1986). The proposed mechanism for this association involves the spread of *Cryptococcus* from the basal cisterns along the perivascular spaces to critical brain structures such as the basal ganglia, lenticular nuclei, internal capsule, and brainstem, which includes the cerebellum. This migration leads to the expansion of perivascular spaces and ensuing vascular compression. Such compression, alongside inflammation, may provoke infarction (Chen et al., 2011a). Furthermore, inflammatory damage to cerebral blood vessels, expressed through vasculitis, vasospasm, and thrombosis, significantly contributes to cerebral infarction among these patients (Mishra et al., 2018; Chen et al., 2011a). A recent case study suggests the obstruction of small veins within the subarachnoid space by inflammatory fibrosis as a novel mechanism underlying stroke in the setting of cryptococcal meningitis (Shimoda et al., 2017). Studies have identified both acute and subacute cerebral infarctions in patients with cryptococcal meningitis (Lan et al., 2001; Chen et al., 2016), with a predilection for the basal ganglia, thalamus, frontal, temporal, and parieto-occipital lobes (Mishra et al., 2018). Within our cohort, 44.7% were found to have lacunar infarcts on initial cranial imaging, indicating ischemic stroke as a predominant feature of intracranial lesions associated with cryptococcal meningitis. The predominance of these lesions in areas such as the basal ganglia, frontal, and parietal lobes corroborates with previous findings (Mishra et al., 2018).

Aseptic meningitis is recognized as the prevailing manifestation of the IRIS in patients with HIV-related cryptococcal meningitis (Haddow et al., 2010). In contrast, cryptococcomas, which appear as mass lesions on imaging, are notably rare. A retrospective study in Italy analyzed 40 patients, of whom 5 developed IRIS; however, only a single case involved a cryptococcoma (Antinori et al., 2009). Moreover, the body of literature on intracranial cryptococcomas, as a consequence of IRIS in the setting of HIV-associated cryptococcal meningitis, is mainly comprised of a few case studies (Breton et al., 2002; Hu et al., 2013; Cattelan et al., 2004). In our study, among the patients who showed progression of intracranial abnormalities post- ART, we identified two instances of cryptococcomas. The diagnosis of these mass lesions was confirmed via surgical pathology.

In this study, approximately one-third of patients exhibited new intracranial lesions or exacerbations of pre-existing ones following ART, with three cases attributed to alternative causes. Among these, the necessity for supplementary medications based on clinical manifestations fell into two categories: mycobacterial infections and cryptococcal immune reconstitution syndrome (C-IRIS). Those suspected of having immune reconstitution syndrome continued with maintenance anti-cryptococcal therapy accompanied by corticosteroids. In patients where an intracranial mycobacterial infection was suspected, some possessed definitive etiological evidence, while others were diagnosed primarily on clinical grounds. For these individuals, in conjunction with prolonged anti-mycobacterial therapy, the majority also received extended corticosteroid treatment, yielding generally favorable outcomes. The clinical picture of CNS mycobacterial infection, characterized by fever, headache, symptoms linked to intracranial lesions, and abnormalities in CSF routine biochemical parameters, closely mirrors that of C-IRIS following ART in HIV patients with a history of cryptococcal meningitis (Haddow et al., 2010), rendering differentiation between the two challenging in the absence of conclusive etiological proof for mycobacterial infection. For these patients, a more aggressive diagnostic and therapeutic approach towards mycobacteria was adopted, resulting in relatively ideal outcomes.

As previously stated, cryptococcal meningitis can prompt intracranial inflammation, resulting in blood vessel damage and potentially leading to vasculitis, which in turn may precipitate cerebral infarction. A prior investigation comparing the clinical characteristics of cryptococcal meningitis patients, both with and without HIV, revealed a notably higher incidence of cerebral infarction among non-HIV-infected patients (P=0.008) (Lee et al., 2011). This incongruity possibly highlights an intensified immune response in HIV-negative individuals, triggering inflammation of vascular walls and thrombotic occurrences (Moosa and Coovadia, 1997). For patients exhibiting pronounced clinical symptoms, the use of glucocorticoids has yielded favorable therapeutic outcomes. Although standardized treatments are lacking for non-life-threatening cases of IRIS, our successful application of proactive glucocorticoid intervention in managing this IRIS manifestation of intracranial vasculitis provides a valuable reference for clinicians and emphasizes the necessity for prospective studies to definitively establish its efficacy.

Our investigation revealed that individuals aged 50 years or older, as well as those presenting with altered consciousness upon admission, were associated with the presence of baseline intracranial lesions. Previous studies have indicated that a significant proportion of our study cohort exhibited intracranial abnormalities related to cerebrovascular issues, thus establishing a strong likelihood of a correlation between advanced age and such lesions. Additionally, the identified association between altered consciousness and pre-existing intracranial lesions resonates closely with clinical intuition and empirical observations.

By categorizing patients based on the development of new intracranial lesions or the progression of existing ones following therapy, we observed that individuals with worsened lesions had significantly higher CSF concentrations of IFN-γ, IL-1Ra, IL-1β, IL-4, IL-5, IL-6, GM-CSF, Eotaxin, and Basic FGF at the four-week treatment mark, compared to those without lesion exacerbation. French et al. previously documented heightened plasma IL-6 levels coinciding with IRIS onset, implicating activated macrophages as a key source (Stone et al., 2002, Stone et al., 2001). In the context of immune equilibrium, the balance between Th17 cells and Regulatory T Cells (Tregs) is paramount; any upset in Th17 cell dynamics can trigger autoimmune disorders. IL-6 is instrumental in this imbalance, as it fosters Th17 differentiation while suppressing Treg differentiation, thereby influencing the initial differentiation of T cells from a homeostatic standpoint (Kimura and Kishimoto, 2010). The detection of a robust Th1-type T cell response alongside heightened pro-inflammatory cytokines, notably IL-6 and TNF-α, in the CSF at IRIS onset, implies a Th1-mediated pro-inflammatory cytokine response in IRIS pathogenesis (Boulware et al., 2010). Our finding provided immunological data for these patients.

This study is subject to several limitations inherent to its retrospective nature. Primarily, our investigation adopts a descriptive approach to analyzing the prevalence of intracranial lesions, diagnostic methodologies, and therapeutic interventions in patients with a history of HIV-associated cryptococcal meningitis, focusing on clinical perspectives. A notable deficiency lies in the absence of standardized reevaluation of imaging findings by specialized radiologists. Although we attempted to classify the etiology of intracranial lesions based on treatment outcomes to the best of our ability, many diagnoses relied heavily on clinical judgment, lacking confirmatory etiological or pathological evidence. Furthermore, the study omitted a case-control comparison of therapeutic protocols, representing another significant limitation. These unresolved issues underscore the necessity for more rigorous scrutiny and resolution through prospective, high-quality, clinical controlled trials in future research endeavors.

In conclusion, intracranial lesions are prevalent in patients diagnosed with HIV-associated cryptococcal meningitis. These lesions can result from pathogens other than *Cryptococcus*, including *Mycobacterium*. Lacunar infarcts are the predominant manifestation of such intracranial lesions among this patient group, with the basal ganglia being the most commonly affected area. Elevated levels of cytokines in the CSF before ART may contribute to the worsening of intracranial lesions in patients with HIV-associated cryptococcal meningitis following ART initiation.

## Data Availability

The raw data supporting the conclusions of this article will be made available by the authors, without undue reservation.
